# Markers of intestinal mucositis to predict blood stream infections at the onset of fever during treatment for childhood acute leukemia

**DOI:** 10.1038/s41375-023-02077-7

**Published:** 2023-11-02

**Authors:** Sarah Weischendorff, Mathias Rathe, Malene Johanne Petersen, Allan Weimann, Christian Enevold, Claus H. Nielsen, Bodil Als-Nielsen, Ulrikka Nygaard, Claus Moser, Klaus Müller

**Affiliations:** 1 Department of Pediatrics and Adolescent Medicine, Rigshospitalet, Copenhagen, Denmark; 2 Institute for Inflammation Research, Center for Rheumatology and Spine Disease, Rigshospitalet, Copenhagen, Denmark; 3 Hans Christian Andersen Children’s Hospital, Odense University Hospital, Odense, Denmark; 4 Department of Clinical Research, University of Southern Denmark, Odense, Denmark; 5 Institute of Clinical Medicine, University of Copenhagen, Copenhagen, Denmark; 6 Department of Clinical Microbiology, Rigshospitalet, Copenhagen, Denmark; 7 Department of Immunology and Microbiology, University of Copenhagen, Copenhagen, Denmark

**Keywords:** Chemotherapy, Medical research

## Abstract

Despite chemotherapy-induced intestinal mucositis being a main risk factor for blood stream infections (BSIs), no studies have investigated mucositis severity to predict BSI at fever onset during acute leukemia treatment. This study prospectively evaluated intestinal mucositis severity in 85 children with acute leukemia, representing 242 febrile episodes (122 with concurrent neutropenia) by measuring plasma levels of citrulline (reflecting enterocyte loss), regenerating islet-derived-protein 3α (REG3α, an intestinal antimicrobial peptide) and CCL20 (a mucosal immune regulatory chemokine) along with the general neutrophil chemo-attractants CXCL1 and CXCL8 at fever onset. BSI was documented in 14% of all febrile episodes and in 20% of the neutropenic febrile episodes. In age-, sex-, diagnosis- and neutrophil count-adjusted analyses, decreasing citrulline levels and increasing REG3α and CCL20 levels were independently associated with increased odds of BSI (OR = 1.6, 1.5 and 1.7 per halving/doubling, all *p* < 0.05). Additionally, higher CXCL1 and CXCL8 levels increased the odds of BSI (OR = 1.8 and 1.7 per doubling, all *p* < 0.0001). All three chemokines showed improved diagnostic accuracy compared to C-reactive protein and procalcitonin. These findings underline the importance of disrupted intestinal integrity as a main risk factor for BSI and suggest that objective markers for monitoring mucositis severity may help predicting BSI at fever onset.

## Introduction

Overall survival of childhood acute leukemia has increased remarkably during the last decades [1–3], but the treatment remains challenged by severe acute toxicities [4]. Chemotherapy-induced neutropenia renders the patients highly susceptible to blood stream infections (BSI) and severe septic progression, accounting for most of the treatment-related mortality [5–7]. Accordingly, broad-spectrum antibiotics are routinely initiated at the onset of fever in patients with neutropenia, even though only 10–30% of these episodes are caused by a verifiable bacterial infection [8–12].

The identification of infections is particularly challenging in patients undergoing high-intensity, myelosuppressive chemotherapy in combination with high-dose glucocorticoids, as this may blur clinical signs of infection. Furthermore, the established biochemical markers of infections, including C-reactive protein (CRP) and procalcitonin (PCT), may also be elevated during non-bacterial infectious toxicities such as drug reactions, inflammatory toxicities, viral infections, and tumor lysis [8–10]. Therefore, more accurate diagnostic tools and better risk management are warranted to improve early diagnosis and to limit the excessive use of antibiotics and the associated adverse effects including multidrug resistant bacteria, nosocomial infections, prolonged hospitalization and microbial dysbiosis [13, 14].

Gastrointestinal mucositis constitutes a main risk factor for BSI due to translocation of bacteria through the disrupted epithelial barrier as supported by studies in patients undergoing hematopoietic stem cell transplantation [15]. Additionally, studies in children with acute lymphoblastic leukemia (ALL) have revealed a close relation between the severity and timing of intestinal mucositis and the occurrence of BSI [16, 17]. However, studies in this patient population are sparse and to date, no studies have evaluated mucositis severity at the onset of fever.

Monitoring intestinal mucositis is challenging due to the lack of validated and robust objective measures of mucositis severity, but recently new, easily measurable markers have been introduced. These include plasma citrulline, a marker of functional enterocytes [18–20], and chemokine (C-C motif) ligand 20 (CCL20), which is a chemokine responsible for mucosal immune regulation, being highly upregulated during mucosal inflammation at various sites to increase the recruitment of lymphocytes [21–23]. Additionally, regenerating islet-derived protein 3α (REG3α), an antimicrobial peptide, can be measured at high levels systemically during gastrointestinal toxicity including inflammatory bowel disease and gastrointestinal acute graft-versus-host disease [24–26]. Nevertheless, to date, no studies have evaluated these markers as a diagnostic approach at the onset of fever to predict BSI in patients undergoing chemotherapy.

The aim of this study was to determine intestinal mucositis severity during febrile episodes based on the hypothesis that more severe intestinal mucositis constituted an increased risk of BSI and that objective markers of mucositis could serve as a diagnostic approach, regardless of the presence of neutropenia.

We investigated quantitative markers of mucositis including citrulline, CCL20 and REG3α in children with acute leukemia at the onset of fever. Additionally, the study examined markers of neutrophil chemotaxis (chemokine (C-X-C motif) ligand 1 and 8; CXCL1 and CXCL8, respectively), which have previously been independently associated with both mucositis and BSI [27–29].

## Methods

### Study population

A total of 85 children (1–18 years) representing 242 febrile episodes during treatment for either acute lymphoblastic leukemia (ALL) or acute myeloid leukemia (AML) at the Department of Pediatrics, Rigshospitalet, Copenhagen, Denmark were enrolled between June 2019 and December 2021. Neutropenia (defined as an absolute neutrophil count (ANC) < 0.5 × 10^9^ cells/l or ANC < 1.0 × 10^9^ cells/l with decreasing levels to <0.5 × 10^9^ cells/l within 2 days) was observed in 122 of the 242 episodes. Fever was defined as a single temperature measurement ≥38.5 °C or sustained temperature measurements ≥38.0 °C for more than 1 h.

Patient characteristics are listed in Table 1. All patients received trimethoprim-sulfamethoxazole twice weekly as prophylaxis for *Pneumocystis jirovecii* infection, while patients with AML or biphenotypical leukemia also typically received voriconazole or equal alternatives as standard antifungal prophylaxis.

**Table 1:** Patient characteristics.

Characteristic	All patients	Patients with neutropenia
Number of patients, no. (%)	85 (100)	63 (100)
Number of males, no. (%)	54 (64)	39 (62)
Age at diagnosis, years (range)	4.0 (1.0–16.5)	4.2 (1.0–16.5)
Diagnosis, no. (%)		
Pre-B ALL	61 (72)	40 (63)
Ph+ ALL	5 (6)	5 (8)
T-ALL	7 (8)	6 (10)
AML	9 (11)	9 (14)
Biphenotypical acute leukemia	3 (3)	3 (5)
Time from diagnosis/relapse, days (range)	87 (6–870)	78 (6–770)
Treatment protocol, no. (%)		
NOPHO ALL 2008	18 (21)	8 (13)
ALLTogether1	44 (52)	35 (55)
NOPHO-DBH AML 2012	8 (9)	8 (13)
EsPhALL	5 (6)	4 (6)
Other^a^	10 (12)	8 (13)

*ALL* acute lymphoblastic leukemia, *AML* acute myeloid leukemia, *EsPhALL* Philadelphia-chromosome-positive acute lymphoblastic leukemia, *NOPHO* Nordic Society for Pediatric Hematology and Oncology, *DBH* Belgian Society of Pediatric Hematology Oncology, The Dutch Childhood Oncology Group, Estonia, and Hong Kong.

^a^Including relapse protocols and iBFM AMBI2018.

### Blood stream infections

Blood for cultures were collected during all febrile episodes from the patients’ central venous catheter (CVC). For patients with a bodyweight <20 kg, 10 ml of blood for one aerobic tube was drawn, and for patients >20 kg, both an aerobic (drawn first) and an anaerobic tube with 10 ml of blood were collected. Microbial identification was performed at the discretion of the regional diagnostic medical microbiological laboratory, and information regarding BSI was collected from the patients’ medical records. The occurrence of BSI was defined as the isolation of one or more bacterial or fungal pathogens from the blood culture. However, two positive blood cultures taken either simultaneously or within 72 h with identical species were required for common skin contaminants including coagulase-negative staphylococci (CoNS), *Cutibacterium acnes* (previously *Propionibacterium acnes*), Micrococcus species, and Corynebacterium species (except *Corynebacterium jeikeium* and *Corynebacterium diphtheria*). BSI with two or more pathogens isolated in the same blood culture or in different blood cultures taken simultaneously were considered polymicrobial.

### Laboratory analyses

EDTA anti-coagulated blood was collected at each episode immediately upon the onset of fever. Plasma was isolated by centrifugation at 2000 × *g* for 10 min and stored at −80 °C within 2 h after sample collection.

Plasma citrulline concentrations were determined by liquid chromatography-tandem mass spectrometry using an ExionLC AD Ultra-High-Performance Liquid Chromatography system coupled to a Sciex QTRAP 6500+ mass spectrometer (AB Sciex, Framingham, MA, USA) with L-citrulline (4,4,5,5-D4) (Cambridge Isotope Laboratories, MA, USA) as the internal standard. Further details can be found in Supplementary Methods.

Plasma levels of CXCL8, CXCL1 and CCL20 were measured using the Bio-Plex Pro Human Chemokine Assay (Bio-Rad, Hercules, CA, USA) with a plasma dilution factor of 1:2 on the Luminex platform (Luminex Corporation, Austin, TX, USA) according to the manufacturer’s instructions.

Plasma levels of REG3α (only analyzed in the 122 samples with neutropenia) were measured by enzyme-linked immunosorbent assay (ELISA) using the Ab-Match Assembly Human PAP1 kit with the Ab-Match Universal kit (MBL International, Woburn, MA, USA), according to the manufacturer’s protocol with a plasma dilution of 1:10.

Plasma CRP, PCT and ANC were measured as part of the clinical routine for all patients. The reported values correspond to the initial measurement taken upon the recognition of fever. CRP was analyzed by an automated immunoturbidimetric assay using the cobas c 702 module (Roche, Rotkreuz, Switzerland), PCT by an electrochemiluminescence immunoassay using the Elecsys BRAHMS PCT assay (Roche Diagnostics Corporation, IN, USA), and ANC by flow cytometry (Sysmex XN, Norderstedt, Germany).

### Statistics

To account for the intraindividual correlation among potentially repeating fever episodes for each patient, a mixed model repeated measures analysis with a compound symmetry covariance structure was used to compare levels of citrulline, CCL20, CXCL1, CXCL8 and REG3α between groups. All variables were log2 transformed due to skewed distributions. In addition, a generalized estimating equation with a compound symmetry covariance structure for within-subject dependencies was used for dichotomized outcomes. Fisher’s exact probability test was used for comparing dichotomized outcomes between groups.

Determination of the sensitivity and specificity for each marker was derived from the receiver operation characteristic (ROC) curve, ignoring the correlation of repeated measurements. The cut-off value was determined by the minimum distance from the left-upper corner of the unit square (sensitivity and specificity equal to 1.0).

Continuous variables are summarized as median (range) unless otherwise stated, and a two-sided *p* value < 0.05 was considered statistically significant. All analyses were performed using the statistical software SAS version 9.4 (SAS Institute, Cary, NC, USA).

## Results

### Febrile episodes and blood stream infections

The median number of febrile episodes per patient was 2 (1–16), and a total number of 59 patients (69%) had two or more episodes. The number of febrile episodes was not associated with either sex, age, or diagnosis.

In 35 of the 242 febrile episodes (14%), BSI was documented by a positive blood culture. In the subgroup of patients with accompanying neutropenia (122 febrile episodes), a BSI was confirmed in 24 of the episodes (20%). Accordingly, only 11 BSI episodes were confirmed in the 120 non-neutropenic febrile episodes (9%). Six patients were represented by two separate BSI episodes. The presence of neutropenia was associated with increased the risk of BSI (OR = 2.35, 95% CI: 1.14–4.82, *p* = 0.020).

Twenty-two different pathogens accounting for a total of 50 positive isolates were identified (Table 2). Eleven BSI episodes were polymicrobial.

**Table 2:** Bacterial and fungal pathogens isolated in patients with bacteremia.

	All patients	Patients with neutropenia
**Positive isolates, no (%)**	**50 (100)**	**25 (100)**
**Gram-positive**	**18 (34)**	**9 (36)**
*Staphylococcus aureus*	4 (8)	2 (8)
Coagulase-negative Staphylococci	4 (8)	2 (8)
* Staphylococcus epidermidis*	4 (8)	2 (8)
Viridans group Streptococci	4 (8)	1 (4)
*Streptococcus mitis* group	3 (6)	0 (0)
*Streptococcus salivarius group*	1 (2)	1 (4)
*Enterococcus faecalis*	1 (2)	0 (0)
*Enterococcus faecium*	2 (4)	2 (8)
*Enterococcus casseliflavus*	1 (2)	0 (0)
*Bacillus cereus*	2 (4)	2 (8)
**Gram-negative**	**32 (60)**	**16 (64)**
Unclassified Gram-negative bacilli	1 (2)	0 (0)
*Acinetobacter pittii*	1 (2)	0 (0)
*Elizabethkingia meningoseptica*	1 (2)	0 (0)
*Pseudomonas species*	2 (4)	0 (0)
*Pseudomonas aeruginosa*	5 (9)	4 (16)
*Pseudomonas putida*	1 (2)	0 (0)
*Pseudomonas stutzeri*	2(4)	1 (4)
*Moraxella osloensis*	1 (2)	1 (4)
*Neisseria subflava*	1 (2)	0 (0)
*Stenotrophomonas maltophilia*	4 (8)	1 (4)
*Enterobacter cloacae*	2 (4)	1 (4)
*Escherichia coli*	3 (6)	3 (12)
*Citrobacter Braakii*	1 (2)	1 (4)
*Klebsiella pneumonia*	7 (13)	5 (20)

The occurrence of BSI was not associated with sex or age, but patients with AML or biphenotypical acute leukemia tended to have a higher prevalence of BSI as compared to patients with ALL during the study period (6/12 (50%) vs. 17/73 (23%), *p* = 0.078).

### Markers of intestinal mucositis in relation to BSI occurrence

Febrile patients with BSI had significantly lower levels of citrulline (15.7 µmol/l (1.3–30.4) vs. 19.4 µmol/l (1.1–61.0), *p* = 0.0094) and increased levels of CCL20 (70.1 pg/ml (1.3–1797.4) vs. 15.6 pg/ml (1.1–1073.1), *p* < 0.0001) compared to febrile patients without BSI.

**Fig. 1:**
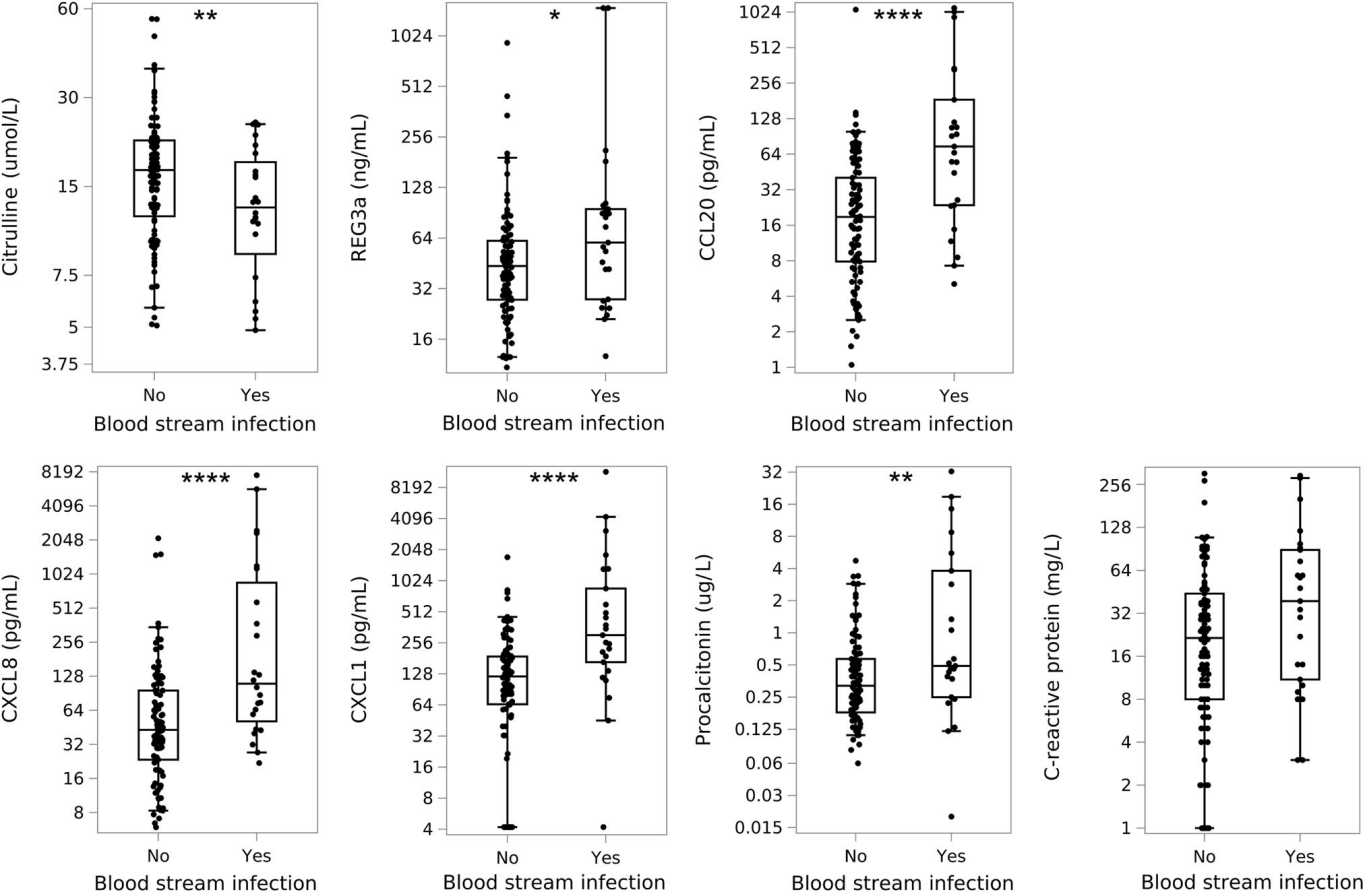
Plasma levels of citrulline, REG3α, CCL20, CXCL8, CXCL1, procalcitonin and C-reactive protein during febrile neutropenia in patients with and without blood stream infections. Boxes show the median level with 25th and 75th percentiles; whiskers represent the 5th and 95th percentiles. Asterisks represent comparison between groups. Note: these plots illustrate the raw data, but statistical analyses accounted for repeating fever episodes among patients. *p < 0.05, **p < 0.01, ***p < 0.001, ***p < 0.0001.

When investigating neutropenic febrile episodes alone, these associations were even more pronounced for citrulline and CCL20. In addition, REG3α was significantly elevated in patients with BSI (Fig. 1).

The risk of BSI increased with decreasing citrulline levels and increasing levels of CCL20 and REG3α. In multivariate analyses adjusted for sex, age, neutropenia, and diagnosis these associations remained significant (Table 3).

**Table 3:** Risk of blood stream infection in univariate- and multivariate analyses.

	Univariate analysis			Multivariate analysis^a^		
Parameter	OR	95% CI	*p* value	OR	95% CI	*p* value
Citrulline	1.8^b^	1.2–2.6	0.0037	1.6^b^	1.1–2.4	0.014
CCL20	1.6	1.3–2.0	<0.0001	1.7	1.3–2.2	<0.0001
REG3α	1.5	1.1–2.0	0.010	1.5	1.01–2.2	0.045
CXCL8	1.5	1.3–1.8	<0.0001	1.7	1.4–2.0	<0.0001
CXCL1	1.5	1.3–1.9	<0.0001	1.8	1.4–2.2	<0.0001
CRP	1.2	1.02–1.5	0.034	1.2	0.9–1.6	0.19
PCT	1.4	1.2–1.8	0.0009	1.4	1.2–1.8	0.0012

*OR* odds ratio, *CI* confidence interval, *REG3α* Regenerating Islet-Derived Protein 3α, *IL-8* interleukin 8, *CRP* C-reactive protein, *PCT* procalcitonin.

^a^Adjusted for sex, age, presence of neutropenia, and diagnosis (acute lymphoblastic leukemia vs. acute myeloid leukemia/biphenotypical leukemia).

^b^OR for citrulline is calculated as per halving in contrast the other parameters, where OR is calculated as per doubling.

### Markers of neutrophil chemotaxis

Both CXCL8 and CXCL1 were significantly increased in patients with BSI compared to those without BSI (87.5 pg/ml (2.1–7640) vs. 18.2 pg/ml (1.5–2117.1), *p* < 0.0001 and 216.2 pg/ml (4.2–12,114.2) vs. 24.2 pg/ml (4.2–1726.3), *p* < 0.0001, respectively). Similarly, elevated CXCL8 and CXCL1 levels were also demonstrated in patients with BSI when only investigating neutropenic febrile episodes as well (Fig. 1).

High levels of CXCL8 and CXCL1 were associated with increased risk of BSI both in univariate and multivariate analyses adjusted for sex, age, neutropenia, and diagnosis (Table 3).

### C-reactive protein and procalcitonin

At the onset of fever, CRP levels were slightly increased in BSI episodes compared to non-BSI-related febrile episodes (22.0 mg/l (1.0–295) vs. 14 mg/l (1.0–305), *p* = 0.045), but this was insignificant for the subgroup with concurrent neutropenia (Fig. 1).

PCT was higher in BSI-related febrile episodes than non-BSI febrile episodes (0.50 µg/l (0.02–32.5) vs. 0.29 µg/l (0.06–27.8), *p* < 0.0001), and this also applied when investigating neutropenic febrile episodes alone (Fig. 1).

Elevated levels of both CRP and PCT were associated with an increased risk of BSI in univariate analyses, but only PCT remained significant in multivariate analyses adjusting for sex, age, neutropenia, and diagnosis (Table 3).

### Evaluation of the diagnostic accuracy for predicting blood stream infections

**Fig. 2:**
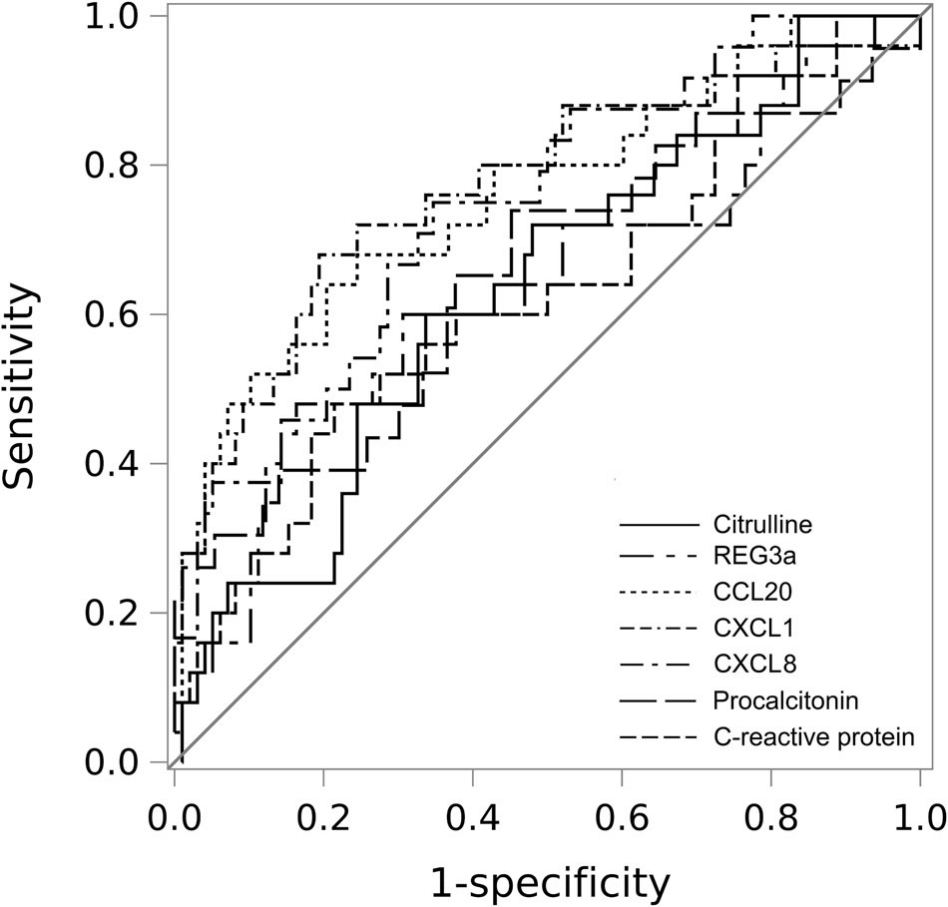
Receiver operating characteristic (ROC)-curves for citrulline, REG3α, CCL20, CXCL1, CXCL8, procalcitonin, and C-reactive protein in patients with febrile neutropenia. The reference line (gray) represents random performance.

ROC curves were established for each univariate model (Fig. 2), estimating the sensitivity and specificity for each parameter at the optimal cut-off value for maximized diagnostic accuracy (Table 4). Furthermore, Supplementary Table 1 details cut-off values for each parameter requiring a reasonable sensitivity of ≥0.75.

**Table 4:** Diagnostic accuracy for detecting blood stream infections.

Parameter	ROC AUC (95% CI)	Cut-off value	Sensitivity	Specificity
All febrile episodes				
Citrulline	0.60 (0.50–0.70)	16.8 µmol/l	0.59	0.58
CCL20	0.73 (0.62–0.84)	40.2 pg/ml	0.65	0.79
CXCL8	0.75 (0.65–0.85)	60.0 pg/ml	0.64	0.80
CXCL1	0.78 (0.68–0.89)	110.6 pg/ml	0.74	0.73
CRP	0.61 (0.50–0.72)	31.0 mg/l	0.45	0.73
PCT	0.66 (0.55–0.78)	0.37 µg/l	0.72	0.59
Neutropenic febrile episodes				
Citrulline	0.65 (0.52–0.77)	13.7 µmol/l	0.61	0.67
CCL20	0.78 (0.66–0.89)	44.5 pg/ml	0.70	0.76
REG3α	0.64 (0.51–0.77)	62.0 ng/ml	0.57	0.70
CXCL8	0.75 (0.63–0.86)	59.0 pg/ml	0.77	0.66
CXCL1	0.77 (0.66–0.89)	189.5 pg/ml	0.74	0.76
CRP	0.62 (0.48–0.76)	34.0 mg/l	0.57	0.67
PCT	0.64 (0.50–0.79)	0.37 µg/l	0.71	0.54

When combining the cut-off value for each parameter with the cut-off value for PCT and CRP in the group of patients with febrile neutropenia, a significant improvement in sensitivity was achieved (0.91 for citrulline, CCL20 and CXCL8 and 0.96 for REG3α and CXCL1 with specificities of 0.27 for citrulline and CXCL8, 0.31 for REG3α and 0.29 for CCL20 and CXCL1, respectively). Including all patients, the combinations reached a sensitivity of 0.85 for CCL20, CXCL1 and CXCL8 (specificities of 0.38, 0.36 and 0.39, respectively) and 0.88 for citrulline (specificity of 0.26).

## Discussion

Improved strategies are needed to promote a more targeted use of antimicrobial therapy without compromising safety in children undergoing chemotherapy. This likely requires a comprehensive, individual approach with effective risk stratification and better diagnostic tools to assist decisions concerning the initiation and duration of antibiotic treatment.

In the present study, we investigated if markers of intestinal mucositis and neutrophil chemotaxis could help differentiate between BSI and non-BSI-related fever episodes in children undergoing treatment for acute leukemia.

We found significant differences in citrulline, REG3α, and CCL20 in patients with BSI, indicating more severe intestinal mucositis in BSI-related fever episodes, emphasizing the serious implications of bacterial translocation through an injured intestinal epithelium. Moreover, the data suggest that mucositis and neutropenia are independent risk-factors for BSI. These results not only validate previous findings in both pediatric and adult patients with hematological cancer, suggesting that citrulline and CCL20 could be valuable in identifying individuals at higher risk of developing BSI during chemotherapy [16, 30–33], but more importantly, they also propose that markers of mucositis can serve as predictive indicators for BSI when patients are admitted with fever.

The significance of a compromised gut barrier integrity as a main site of bacterial translocation was further supported by the fact that many of the BSI episodes were caused by bacteria most likely originating from the oral cavity, the pharynx or the gut, including *Escherichia coli*, *Klebsiella* species, *Enterococcus* species, Viridans group streptococci, *Enterobacter cloacae*, *Citrobacter Braakii*, *Moraxella osloensis* and *Neisseria subflava*. Previous studies found positive cultures with Gram-positive bacteremia (with especially CoNS and *Staphylococcus aureus*) relatively more abundant [34–36], but a huge effort has been made in recent years to limit central line associated infections in children with cancer, which could explain this development [37–40]. Furthermore, our strict definition requiring at least two positive cultures for common contaminants (CoNS, micrococcus species etc.) has likely limited false positives.

In addition to citrulline, CCL20 and REG3α, all of which have been closely related to intestinal damage and inflammation, we also measured the neutrophil chemo-attractants CXCL1 and CXCL8. While CXCL1 has not previously been measured during febrile episodes in children with leukemia, CXCL8 has been extensively studied [27, 41–43]. These chemokines are closely related, originating from similar cellular sources, and being upregulated in response to same immunological stimuli [44]. Both markers were significantly elevated in patients with BSI-related febrile episodes as compared to non-BSI-related febrile episodes, confirming previous findings demonstrating increased CXCL1 and CXCL8 levels prior to both clinical symptoms and increasing CRP levels during BSI in children with ALL [29].

For citrulline and REG3α the diagnostic accuracy was comparable to CRP, while CCL20, CXCL1 and CXCL8 showed better diagnostic accuracy than CRP and PCT. This may likely be related to the fact that febrile mucositis is observed also in the absence of a documented infection [45], and as BSI episodes may also be caused by dissemination of infections unrelated to gut translocation. Accordingly, the superior diagnostic ability of CCL20 in these patients as compared to citrulline and REG3α could be explained by a release of this chemokine at other mucosal linings including in the respiratory tract.

While both CXCL1 and CXCL8 have been associated with intestinal mucositis, reflecting increased neutrophil recruitment to drive mucosal inflammation [28, 29, 46], both chemokines are more generally upregulated during inflammation with the ability to capture all sources of BSI [44, 47]. This is supported by several studies, suggesting CXCL8 as the most accurate diagnostic marker to differentiate between infectious and non-infectious neutropenic fever episodes during childhood cancer treatment [27, 42]. Nevertheless, defining a reasonable sensitivity level becomes particularly pertinent in the context of highly susceptible patients as in the current setting. Therefore, the cut-off value that maximizes diagnostic accuracy may not align with a clinical operational cut-off value. It is most likely that we will need to combine multiple risk factors to enhance predictive capabilities.

Interestingly, all the studied markers showed comparable ability to detect BSI-related febrile episodes when combined with the currently implemented diagnostic tools (CRP and PCT) with a sensitivity of >0.90 for neutropenic patients. This underlines the potential for risk stratification by biomarkers reflecting the early intestinal injury that initiates and propagates the inflammatory, antimicrobial defense cascade, as previously suggested [16, 29]. The relatively lower specificity observed along this high sensitivity should be considered in the context of current guidelines, which recommend the initiation of empiric intravenous, broad-spectrum antibiotics for all high-risk patients at the onset of neutropenic fever, even though less than half of these patients are ultimately diagnosed with an infection. Even with this limitation, the ability to exclude severe infections in 25% of all febrile neutropenic episodes has the potential to enhance safety when considering early antibiotic cessation. Importantly, these findings are highly exploratory and need to be validated before final conclusions.

The main strength of this study is the relatively large, homogenous patient cohort of children with acute leukemia and the inclusion of all febrile episodes as well as the coordinated collection of plasma samples at the first clinical assessment after the onset of symptoms. The strict guidelines requiring blood cultures for all patients presenting with fever makes the outcome of BSI particularly robust, as no missing information is present. However, the large variation in the collection of other microbiological tests and diagnostic imaging, including cultures from alternative foci (urine, tracheal aspirate, stool), and nasopharyngeal swabs for virus detection prevented an appropriate characterization of blood culture negative focal infections due to the large number of untested individuals.

Other important limitations include the observational nature of the study, limiting our ability to infer causal relations and to control for possible interfering parameters such as the use of antibiotics. Moreover, the study would have benefited from repeated measurements of the markers to determine their kinetics and to evaluate the diagnostic accuracy after, e.g., 24–48 h of fever onset. Additionally, having an individual baseline level for citrulline to reflect relative changes during chemotherapy might be of importance in the pediatric setting.

In conclusion, there is growing evidence supporting the present markers as robust, objective and minimally invasive tools to evaluate mucositis severity, which definitely increases feasibility, validity and reproducibility in both research and clinical settings. Our results further indicate that a dysregulated intestinal barrier is a significant risk factor for invasive infections and that monitoring intestinal mucositis by quantitative markers may serve as a potential diagnostic approach to detect BSI-related fever episodes. These findings also emphasize the importance of protecting the gastrointestinal barrier during chemotherapy to reduce infectious morbidity, which merits increased clinical and scientific priority. Controlled, clinical trials are needed to investigate whether these markers can be used clinically to guide antimicrobial therapy.

### Supplementary information

Quantitation of plasma citrulline

Supplementary Table 1

## Data Availability

The data supporting these findings are available from the corresponding author upon reasonable request and after regulatory approval.

## References

[1] Hunger SP, Mullighan CG (2015). Acute lymphoblastic leukemia in children. N Engl J Med.

[2] Gatta G, Botta L, Rossi S, Aareleid T, Bielska-Lasota M, Clavel J (2014). Childhood cancer survival in Europe 1999-2007: results of EUROCARE-5—a population-based study. Lancet Oncol.

[3] Zwaan CM, Kolb EA, Reinhardt D, Abrahamsson J, Adachi S, Aplenc R (2015). Collaborative efforts driving progress in pediatric acute myeloid leukemia. J Clin Oncol.

[4] Toft N, Birgens H, Abrahamsson J, Griškevičius L, Hallböök H, Heyman M (2016). Toxicity profile and treatment delays in NOPHO ALL2008-comparing adults and children with Philadelphia chromosome-negative acute lymphoblastic leukemia. Eur J Haematol.

[5] Inaba H, Pei D, Wolf J, Howard SC, Hayden RT, Go M (2017). Infection-related complications during treatment for childhood acute lymphoblastic leukemia. Ann Oncol.

[6] BODEY GP, Buckley M, Sathe YS, Freireich EJ (1966). Quantitative relationships between circulating leukocytes and infection in patients with acute leukemia. Ann Intern Med.

[7] Afzal S, Ethier MC, Dupuis LL, Tang L, Punnett AS, Richardson SE (2009). Risk factors for infection-related outcomes during induction therapy for childhood acute lymphoblastic leukemia. Pediatr Infect Dis J.

[8] Lehrnbecher T, Robinson P, Fisher B, Alexander S, Ammann RA, Beauchemin M (2017). Guideline for the management of fever and neutropenia in children with cancer and hematopoietic stem-cell transplantation recipients: 2017 update. J Clin Oncol.

[9] Robinson PD, Lehrnbecher T, Phillips R, Lee Dupuis L, Sung L (2016). Strategies for empiric management of pediatric fever and neutropenia in patients with cancer and hematopoietic stem-cell transplantation recipients: a systematic review of randomized trials. J Clin Oncol.

[10] Castagnola E, Fontana V, Caviglia I, Caruso S, Faraci M, Fioredda F (2007). A prospective study on the epidemiology of febrile episodes during chemotherapy-induced neutropenia in children with cancer or after hemopoietic stem cell transplantation. Clin Infect Dis.

[11] Flowers CR, Seidenfeld J, Bow EJ, Karten C, Gleason C, Hawley DK (2013). Antimicrobial prophylaxis and outpatient management of fever and neutropenia in adults treated for malignancy: American Society of Clinical Oncology Clinical Practice Guideline. J Clin Oncol.

[12] Falagas ME, Vardakas KZ, Samonis G (2008). Decreasing the incidence and impact of infections in neutropenic patients: evidence from meta-analyses of randomized controlled trials. Curr Med Res Opin.

[13] El-Mahallawy HA, El-Wakil M, Moneer MM, Shalaby L (2011). Antibiotic resistance is associated with longer bacteremic episodes and worse outcome in febrile neutropenic children with cancer. Pediatr Blood Cancer.

[14] Kamboj M, Sepkowitz KA (2009). Nosocomial infections in patients with cancer. Lancet Oncol.

[15] Blijlevens NMA, de Mooij CEM (2023). Mucositis and infection in hematology patients. Int J Mol Sci.

[16] De Pietri S, Weischendorff S, Rathe M, Leth Frandsen T, Hasle H, Nersting J, et al. Gastrointestinal barrier integrity and mucosal inflammation as risk factors of blood stream infections in children treated for acute lymphoblastic leukaemia. Int J Cancer. 2023;153:1635–42. 10.1002/ijc.34639 37387257

[17] De Pietri S, Frandsen TL, Christensen M, Grell K, Rathe M, Müller K (2021). Citrulline as a biomarker of bacteraemia during induction treatment for childhood acute lymphoblastic leukaemia. Pediatr Blood Cancer.

[18] Fragkos KC, Forbes A (2018). Citrulline as a marker of intestinal function and absorption in clinical settings: a systematic review and meta-analysis. United Eur Gastroenterol J.

[19] van Vliet MJ, Tissing WJE, Rings EHHM, Koetse HA, Stellaard F, Kamps WA (2009). Citrulline as a marker for chemotherapy induced mucosal barrier injury in pediatric patients. Pediatr Blood Cancer.

[20] Lugtens JCHW, Blijlevens NMA, Deutz NEP, Donnelly JP, Lambin P, De Pauw BE (2005). Monitoring myeloablative therapy-induced small bowel toxicity by serum citrulline concentration: a comparison with sugar permeability tests. Cancer.

[21] Kaser A, Ludwiczek O, Holzmann S, Moschen AR, Weiss G, Enrich B (2004). Increased expression of CCL20 in human inflammatory bowel disease. J Clin Immunol.

[22] Trivedi PJ, Adams DH (2018). Chemokines and chemokine receptors as therapeutic targets in inflammatory bowel disease; pitfalls and promise. J Crohns Colitis.

[23] Allodi M, Giorgio C, Incerti M, Corradi D, Flammini L, Ballabeni V (2023). Probing the effects of MR120 in preclinical chronic colitis: a first-in-class anti-IBD agent targeting the CCL20/CCR6 axis. Eur J Pharm.

[24] Sun C, Wang X, Hui Y, Fukui H, Wang B, Miwa H (2021). The potential role of REG family proteins in inflammatory and inflammation-associated diseases of the gastrointestinal tract. Int J Mol Sci.

[25] Isnard S, Ramendra R, Dupuy FP, Lin J, Fombuena B, Kokinov N (2020). Plasma levels of C-type lectin REG3α and gut damage in people with human immunodeficiency virus. J Infect Dis.

[26] Ferrara JLM, Harris AC, Greenson JK, Braun TM, Holler E, Teshima T (2011). Regenerating islet-derived 3-alpha is a biomarker of gastrointestinal graft-versus-host disease. Blood.

[27] Arif T, Phillips RS (2019). Updated systematic review and meta-analysis of the predictive value of serum biomarkers in the assessment and management of fever during neutropenia in children with cancer. Pediatr Blood Cancer.

[28] Miedema KGE, De Bont ESJM, Elferink RFMO, Van Vliet MJ, Nijhuis CSMO, Kamps WA (2011). The diagnostic value of CRP, IL-8, PCT, and sTREM-1 in the detection of bacterial infections in pediatric oncology patients with febrile neutropenia. Support Care Cancer.

[29] Weischendorff S, De Pietri S, Rathe M, Frandsen TL, Hasle H, Nielsen CH, et al. Markers of neutrophil chemotaxis for identification of blood stream infections in children with acute lymphoblastic leukemia undergoing induction treatment. Eur J Haematol. 2023;110:762–71. 10.1111/ejh.13962 36950865

[30] de Mooij CEM, van der Velden WJFM, de Haan AFJ, Fazel S, van Groningen LFJ, Blijlevens NMA (2022). Grading bloodstream infection risk using citrulline as a biomarker of intestinal mucositis in patients receiving intensive therapy. Bone Marrow Transplant.

[31] Herbers AHE, Blijlevens NMA, Donnelly JP, de Witte TJM (2008). Bacteraemia coincides with low citrulline concentrations after high-dose melphalan in autologous HSCT recipients. Bone Marrow Transplant.

[32] Herbers AHE, De Haan AFJ, Van der Velden WJFM, Donnelly JP, Blijlevens NMA (2014). Mucositis not neutropenia determines bacteremia among hematopoietic stem cell transplant recipients. Transpl Infect Dis.

[33] van der Velden WJFM, Herbers AHE, Feuth T, Schaap NPM, Donnelly JP, Blijlevens NMA (2010). Intestinal damage determines the inflammatory response and early complications in patients receiving conditioning for a stem cell transplantation. PLoS ONE.

[34] Lausen B, Schmiegelow K, Andreassen B, Madsen HO, Garred P (2006). Infections during induction therapy of childhood acute lymphoblastic leukemia—no association to mannose-binding lectin deficiency. Eur J Haematol.

[35] Rungoe C, Malchau EL, Larsen LN, Schroeder H (2010). Infections during induction therapy for children with acute lymphoblastic leukemia. The role of sulfamethoxazole-trimethoprim (SMX-TMP) prophylaxis. Pediatr Blood Cancer.

[36] Bergmann K, Hasle H, Asdahl P, Handrup MM, Wehner PS, Rosthøj S (2016). Central venous catheters and bloodstream infection during induction therapy in children with acute lymphoblastic Leukemia. J Pediatr Hematol Oncol.

[37] Owings A, Graves JB, Johnson S, Gilliam C, Gipson M, Hakim H (2018). Leadership line care rounds: application of the engage, educate, execute, and evaluate improvement model for the prevention of central line-associated bloodstream infections in children with cancer. Am J Infect Control.

[38] Bundy DG, Gaur AH, Billett AL, He B, Colantuoni EA, Miller MR (2014). Preventing CLABSIS among pediatric hematology/oncology inpatients: national collaborative results. Pediatrics.

[39] Beville ASM, Heipel D, Vanhoozer G, Bailey P (2021). Reducing central line associated bloodstream infections (CLABSIs) by reducing central line days. Curr Infect Dis Rep.

[40] Dandoy CE, Hausfeld J, Flesch L, Hawkins D, Demmel K, Best D (2016). Rapid cycle development of a multifactorial intervention achieved sustained reductions in central line-associated bloodstream infections in haematology oncology units at a children’s hospital: a time series analysis. BMJ Qual Saf.

[41] Phillips B (2022). Prospective cohort study of the predictive value of inflammatory biomarkers over clinical variables in children and young people with cancer presenting with fever and neutropenia. F1000Research.

[42] Phillips RS, Wade R, Lehrnbecher T, Stewart LA, Sutton AJ (2012). Systematic review and meta-analysis of the value of initial biomarkers in predicting adverse outcome in febrile neutropenic episodes in children and young people with cancer. BMC Med.

[43] Haeusler GM, Carlesse F, Phillips RS (2013). An updated systematic review and meta-analysis of the predictive value of serum biomarkers in the assessment of fever during neutropenia in children with cancer. Pediatr Infect Dis J.

[44] Petri B, Sanz MJ (2018). Neutrophil chemotaxis. Cell Tissue Res.

[45] van der Velden WJFM, Herbers AHE, Netea MG, Blijlevens NMA (2014). Mucosal barrier injury, fever and infection in neutropenic patients with cancer: introducing the paradigm febrile mucositis. Br J Haematol.

[46] Wardill HR, de Mooij CEM, Da Silva Ferreira AR, Havinga H, Harmsen HJM, van der Velden WJFM (2022). Supporting the gastrointestinal microenvironment during high-dose chemotherapy and stem cell transplantation by inhibiting IL-1 signaling with anakinra. Sci Rep.

[47] Kobayashi Y (2008). The role of chemokines in neutrophil biology. Front Biosci.

