# Supplementary material for: Markers of intestinal mucositis to predict blood stream infections at the onset of fever during treatment for childhood acute leukemia
Source: Leukemia. 2023 Nov 2;38(1):14–20. doi: 10.1038/s41375-023-02077-7 (PMC10776407; doi:10.1038/s41375-023-02077-7)
Supplement: Supplementary file 1 — Quantitation of plasma citrulline [file 41375_2023_2077_MOESM1_ESM.docx]

**Quantitation of plasma citrulline**

*Sample preparation*

Sample preparation was performed by mixing 10 µL of sample plasma, calibration standards or water (blank) with 900 µL of 90% acetonitrile spiked with internal standard (L-citrulline (4,4,5,5-D4) (Cambridge Isotope Laboratories, MA, USA). After vortex mixing, samples were centrifuged for 15 min at 3220 g, and the supernatants were analysed with liquid chromatography-tandem mass spectrometry (LC-MS/MS). The calibration standards were prepared by dissolving L-citrulline (Sigma-Aldrich, MA, USA) in water and further diluting it in 90% acetonitrile, to obtain final concentrations of 0.25, 0.70, 2.22, 6.66, 20.0 and 60.0 µM, using the surrogate matrix approach due to lack of a blank matrix.

*LC-MS/MS analysis*

LC-MS/MS analysis was performed using an ExionLC AD Ultra-High-Performance Liquid Chromatography (UHPLC) system (AB Sciex, MA, USA), equipped with a thermostatted column compartment (40°C), an AD binary pump, an AD multiplate sample manager and a degasser. The UHPLC system was coupled to a Sciex QTRAP 6500+ mass spectrometer (AB Sciex, MA, USA) with an electrospray ionization probe. Chromatographic separations were performed on an Acquity Ultra-Performance Liquid Chromatography (UPLC) ethylene bridged hybrid amide column (130 Å, 1.7 µm, 2.1 mm x 50 mm) (Waters Corp, Milford, MA, USA) with an injection volume of 1.5 µL. Citrulline was separated using a binary gradient in hydrophilic interaction liquid chromatography (HILIC) mode (mobile phase A: water containing 0.1 % formic acid; Mobile phase B: acetonitrile containing 0.1% formic acid). The gradient was as follows: 0 min: 85% B; 3 min: 60% B; 3.2 min: 35% B; 3.7 min: 35% B; 4.0 min: 85% B.

Ionization was achieved with an ionization spray voltage of 4000 V and an ion source temperature of 400ºC. Citrulline and citrulline-(4,4,5,5-D4) were detected in positive mode by multiple reaction monitoring with *m/z* transitions of 176 → 70 (quantifier) and 176 → 113 (qualifier) for citrulline and 180 → 74 for citrulline-(4,4,5,5-D4), with a dwell time of 200 msec, curtain gas of 20 L/h and collision energies of 30 eV (quantifier) and 21 eV (qualifier) with nitrogen as the collision gas. System operations and data acquisition were controlled by Analyst 1.7.3 software (AB Sciex, MA, USA), and data processing was performed using MulitQuant 3.0.3 software (AB Sciex, MA, USA).

*Linearity, precision, and accuracy*

Linearity was assessed for citrulline in the range 0.25-60.0 µM, with a 1/x weighed linear regression curve, which showed sufficient linearity in the calibration range used. Intra- and inter- day precision was performed using a reversed standard curve approach, with plasma citrulline as internal standard and citrulline-(4,4,5,5-D4) as analyte (lower limit of quantification, medium and high concentrations). Plasma was also spiked with citrulline, to obtain medium and high concentrations, using background subtraction for concentration calculations. All samples were measured on 3 different days, each in quadruplicates, with relative standard deviation (RSD) < 10% and accuracy within ± 7% of the nominal concentrations.
