# Supplementary material for: Markers of intestinal mucositis to predict blood stream infections at the onset of fever during treatment for childhood acute leukemia
Source: Leukemia. 2023 Nov 2;38(1):14–20. doi: 10.1038/s41375-023-02077-7 (PMC10776407; doi:10.1038/s41375-023-02077-7)
Supplement: Supplementary file 2 — Supplementary Table 1 [file 41375_2023_2077_MOESM2_ESM.docx]

SUPPLEMENTARY TABLE 1: Cut-off values for detecting blood stream infections with sensitivities of ≥0.75.

| Parameter | Cut-off value | Sensitivity | Specificity |
| --- | --- | --- | --- |
| All febrile episodes | | | |
| Citrulline | 20.5 µmol/L | 0.75 | 0.36 |
| CCL20 | 14.8 pg/mL | 0.75 | 0.47 |
| CXCL8 | 27.2 pg/mL | 0.77 | 0.59 |
| CXCL1 | 75.0 pg/mL | 0.75 | 0.63 |
| CRP | 8.0 mg/L | 0.80 | 0.35 |
| PCT | 0.25 µg/L | 0.76 | 0.43 |
| Neutropenic febrile episodes | | | |
| Citrulline | 18.1 µmol/L | 0.76 | 0.42 |
| CCL20 | 23.7 pg/mL | 0.76 | 0.58 |
| REG3α | 27.6 ng/mL | 0.76 | 0.26 |
| CXCL8 | 59.0 pg/mL | 0.75 | 0.65 |
| CXCL1 | 166.5 pg/mL | 0.76 | 0.65 |
| CRP | 11.0 mg/L | 0.76 | 0.31 |
| PCT | 18.1 µg/L | 0.76 | 0.42 |
